# New Treatments Are Urgently Needed for Patients With All Primary Ciliary Dyskinesia Genotypes

**DOI:** 10.1002/ppul.71372

**Published:** 2025-11-12

**Authors:** Benjamin Gaston, Amjad Horani, Stephanie Davis, Thomas Ferkol, BreAnna Kinghorn, Margaret Rosenfeld, Adam Shapiro, Evans Machogu, Michael O'Connor, Harriet Holme, Michele Manion

**Affiliations:** ^1^ Indiana University Bloomington Indiana USA; ^2^ Washington University St. Louis Missouri USA; ^3^ University of North Carolina Chapel Hill North Carolina USA; ^4^ Seattle Children's Hospital Seattle Washington USA; ^5^ McGill University Montreal Canada; ^6^ Vanderbilt University Nashville Tennessee USA; ^7^ PCD Research UK; ^8^ PCD Foundation Minneapolis Minnesota USA

To the editor,

We read with interest the letter from Pifferi et al. on lung function decline in patients with primary ciliary dyskinesia (PCD) [1]. We congratulate the authors on the good lung function trajectories achieved by many of their PCD patients. We look forward to hearing more about these therapeutic successes. As PCD providers, parents and patient advocates, however, we are concerned that this study as presented is being interpreted by those in industry who are considering the development of new therapeutic options to suggest that market opportunities among may not be robust among PCD patients with certain genotypes. That is to say, there is the thought among some colleagues in industry that investment in PCD therapies may not be needed or worthwhile. I think the authors would agree that this conclusion is incorrect.

As the authors know, PCD patients with a range of genotypes have lung disease that can progress to respiratory failure [2, 3]. Even among those who do not progress to respiratory failure, patients are chronically affected by daily cough and mucus production; disabling bronchiectasis; disrupted work and sleep; recurring pneumonias; frequent exacerbations; and burdensome yet marginally effective treatment regimens [2, 4]. Additionally, the majority of newborns with PCD experience respiratory distress shortly after birth [2]. Some of those most severely affected during the newborn period may not survive to be included in longitudinal lung function analyses.

And these are just the pulmonary symptoms. Head and neck symptoms are particularly devastating in childhood, and they progress into adulthood. Nonstop rhinorrhea is a daily burden, often accompanied by anosmia. Severe, chronic otitis media is common, with daily purulent otorrhea and hearing loss. Chronic sinusitis and sinus headaches are the norm [4].

Though patients with some genotypes may be less severely affected than others [1], there is variability within the same genotype. Importantly, patients with most PCD genotypes suffer from these daily consequences and are at risk for respiratory failure. Further, PCD appears to be substantially more common than was once thought [5]. Emerging data from large PCD registries are bearing out this unfortunate reality. We are confident that Pifferi and colleagues will agree that effective treatments are urgently needed for PCD patients with a broad range of different genotypes.

## Author Contributions

**Benjamin Gaston:** conceptualization, project administration, resources, writing – original draft, writing – review and editing. **Amjad Horani:** conceptualization, writing – review and editing. **Stephanie Davis:** conceptualization. **Thomas Ferkol:** conceptualization, writing – review and editing. **BreAnna Kinghorn:** conceptualization. **Margaret Rosenfeld:** conceptualization, writing – review and editing. **Adam Shapiro:** conceptualization, writing – review and editing. **Evans Machogu:** conceptualization, writing – review and editing. **Michael O'Connor:** conceptualization, writing – review and editing. **Harriet Holme:** conceptualization, writing – review and editing. **Michele Manion:** conceptualization, writing – review and editing.

## Acknowledgments

The authors have nothing to report.

## Conflicts of Interest

The authors declare no conflicts of interest.

## Data Availability

Data sharing is not applicable to this article as no data sets were generated or analyzed during the current study.
